# Transcriptomic analysis of the stress response to weaning at housing in bovine leukocytes using RNA-seq technology

**DOI:** 10.1186/1471-2164-13-250

**Published:** 2012-06-18

**Authors:** Aran O’Loughlin, David J Lynn, Mark McGee, Sean Doyle, Matthew McCabe, Bernadette Earley

**Affiliations:** 1 Animal and Bioscience Research Department, Animal & Grassland Research and Innovation Centre, Teagasc, Grange, Dunsany, Co. Meath, Ireland; 2 Department of Biology and National Institute for Cellular Biotechnology, National University of Ireland Maynooth, Co. Kildare, Ireland; 3 Livestock Systems Research Department, Animal & Grassland Research and Innovation Centre, Teagasc, Grange, Dunsany, Co. Meath, Ireland

## Abstract

**Background:**

Weaning of beef calves is a necessary husbandry practice and involves separating the calf from its mother, resulting in numerous stressful events including dietary change, social reorganisation and the cessation of the maternal-offspring bond and is often accompanied by housing. While much recent research has focused on the physiological response of the bovine immune system to stress in recent years, little is known about the molecular mechanisms modulating the immune response. Therefore, the objective of this study was to provide new insights into the molecular mechanisms underlying the physiological response to weaning at housing in beef calves using Illumina RNA-seq.

**Results:**

The leukocyte transcriptome was significantly altered for at least 7 days following either housing or weaning at housing. Analysis of differentially expressed genes revealed that four main pathways, cytokine signalling, transmembrane transport, haemostasis and G-protein-coupled receptor (GPRC) signalling were differentially regulated between control and weaned calves and underwent significant transcriptomic alterations in response to weaning stress on day 1, 2 and 7. Of particular note, chemokines, cytokines and integrins were consistently found to be up-regulated on each day following weaning. Evidence for alternative splicing of genes was also detected, indicating a number of genes involved in the innate and adaptive immune response may be alternatively transcribed, including those responsible for toll receptor cascades and T cell receptor signalling.

**Conclusions:**

This study represents the first application of RNA-Seq technology for genomic studies in bovine leukocytes in response to weaning stress. Weaning stress induces the activation of a number of cytokine, chemokine and integrin transcripts and may alter the immune system whereby the ability of a number of cells of the innate and adaptive immune system to locate and destroy pathogens is transcriptionally enhanced. Stress alters the homeostasis of the transcriptomic environment of leukocytes for at least 7 days following weaning, indicating long term effects of stress exposure in the bovine. The identification of gene signature networks that are stress activated provides a mechanistic framework to characterise the multifaceted nature of weaning stress adaptation in beef calves. Thus, capturing subtle transcriptomic changes provides insight into the molecular mechanisms that underlie the physiological response to weaning stress.

## Background

Weaning is a stressful event in the calf’s lifetime with alterations in behaviour [1-4], hormonal mediators of stress [5,6], and immune function [7-11] documented post-weaning. Stress-induced changes in immune function have been documented in cattle with alterations to cell-mediated and humoral immunity having a significant impact on immunocompetence which may increase susceptibility to disease [12-18]. Weaning, together with movement of beef calves from a pasture environment to a housing environment, has been shown to negatively affect total leukocyte, neutrophil and lymphocyte counts, lymphocyte immunophenotypes and the functional activity of neutrophils in beef calves [19]. These authors also reported a neutrophil and lymphocyte immunophenotypic response to housing of calves, indicating that housing can elicit a stress response, even when separated from weaning. More recently, O’Loughlin et al. [10] reported that the expression of pro-inflammatory cytokine genes in leukocytes, including IL-1β, IL-8, IFN-γ and TNFα, were up-regulated, up to 7 days post weaning, in calves that were housed together with their dams for 28 days prior to separation. The molecular mechanisms involved and the role of glucocorticoids in modulating the immune response to weaning at housing, however, remain to be elucidated.

Few studies in any species have sought to characterise the stress response and identify the regulatory input by cells of the immune system, particularly at the transcriptomic level. RNA-seq is a relatively new technique that provides a unique opportunity to deeply sequence the transcriptome of any species with single base resolution and without the need for a known reference genome sequence, allowing unparalleled, highly accurate quantification of differential gene expression, in addition to the identification of novel or unannotated genes, transcripts and alternative splicing events. Furthermore, RNA-seq generates absolute gene expression measurements, providing greater resolution and accuracy than microarrays [20]. Recently, Huang and Khatib [21] published the first bovine study to use RNA-seq, focusing on the transcriptomic landscape of embryos and concluded that this technology was highly suitable for future gene expression studies in the bovine, a conclusion in line with other groups [22]. In this study, a global gene expression approach was used to investigate the response to weaning at housing and to elucidate the key regulatory genes and pathways in beef calves. Thus, capturing subtle transcriptomic changes could provide insight into the molecular mechanisms that underlie the physiological response to stress.

## Methods

All animal procedures performed in this study were conducted under experimental licence from the Irish Department of Health and Children in accordance with the Cruelty to Animals Act 1876 and the European Communities (Amendment of Cruelty to Animals Act 1876) Regulation 2002 and 2005.

### Animal model

**Figure 1:**
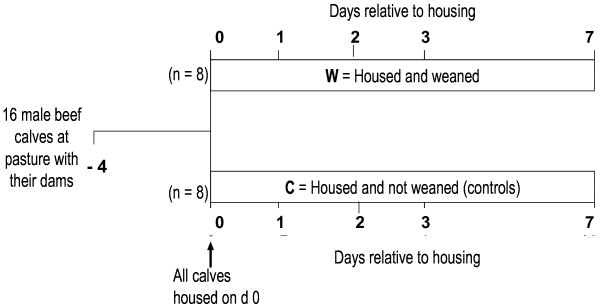
**Experimental design and rectal body temperature recording and blood sampling schedule for beef calves that were housed on day (d) 0, and either (i) weaned (W: n = 8), or (ii) not weaned (controls) (C: n = 8).** Rectal body temperatures were recorded and blood samples were collected on d −4, 0, 1, 2, 3, and 7 relative to housing on d 0. On d 0, samples were obtained prior to housing.

Sixteen clinically healthy, spring-born, single-suckled, intact male Simmental beef calves were used in the study (Figure 1). Calves were born at the Teagasc Grange Research Centre and as part of standard husbandry and research management practices were accustomed to stockpersons and routine handling in the facilities. Calves were rotationally grazed together with their dams on a predominantly perennial ryegrass (*Lolium perenne*) based sward from April until weaning on 20 October, 2009. All calves in the study were immunized 28 days prior to weaning against bovine respiratory syncytial virus (BRSV) and infectious bovine rhinotracheitis (IBR) virus using *Rispoval-3* and *Rispoval-IBR* vaccines (Pfizer Animal Health, Co. Cork, Ireland), respectively. On day (d) 0, all calves were moved to a handling yard where they were assigned to one of two treatments prior to housing: 1), Control calves (C; n = 8; mean weight (s.d) 251.0 (47.8) kg; mean age (s.d.) 199 (34.4) days), were housed in pens with their dam (2 cow-calf pairs per pen) on concrete slatted floors, 2), Weaned calves (W; n = 8; mean weight (s.d.) 250.4 (29.1) kg; mean age (s.d.) 209 (35.4) days), were abruptly separated from their dam and housed in pens with other abruptly weaned calves (4 calves per pen), introducing a social reorganisation component. All pens were equipped with automatic drinkers, had concrete slatted floors and the space allowance in each pen was equal for animals. Calves were offered grass silage *ad libitum* and 1 kg/head/day concentrate feed throughout the study.

### Rectal temperature measurement

Rectal body temperature was recorded using a digital thermometer (Jorgen Kruuse A/S; Model VT-801 BWC Lot No 0701, Marslev, Denmark) on each occasion while the calves were waiting in the holding chute immediately prior to blood sample collection (Figure 1).

### Blood sample collection

Blood was sampled via jugular venipuncture on days −4, 0, 1, 2, 3 and 7 relative to weaning (d 0) (Figure 1). For this procedure, calves were led gently to a holding pen, with a squeeze chute facility and were blood sampled with minimal restraint. Blood sampling was carried out by the same experienced operator on each occasion and the time taken to collect the blood samples was less than 60 s/calf. Blood samples were collected into 1 × 6 mL K_3_Ethylenediaminetetraacetic acid (K_3_EDTA) tubes for haematological analysis, 1 × 8 mL lithium heparin tubes for haptoglobin analysis and 5 × 9 mL acid citrate dextrose (ACD) tubes (Vacuette, Cruinn Diagnostics, Ireland) for leukocyte isolations.

### Haematology variables

Unclotted whole K_3_EDTA blood samples were analysed using an ADVIA haematology analyser (AV ADVIA 2120, Bayer Healthcare, Siemens, UK) equipped with software for bovine blood. Total leukocyte, neutrophil, lymphocyte, eosinophil and monocyte percentage, red blood cell (RBC) number, haemoglobin (HGB), mean corpuscular haemoglobin concentration (MCHC), haematocrit (HCT) percentage and platelet (PLT) number were measured. The neutrophil:lymphocyte (N:L) ratio was also calculated.

### Haptoglobin and cortisol

Plasma was harvested from the lithium heparin anti-coagulated blood tubes following centrifugation at 1600 × *g* at 4 °C for 15 min and stored at −80 °C until analysis. The concentration of plasma haptoglobin was measured using an automatic analyzer (spACE, Alfa Wassermann, Inc., West Caldwell, NJ, USA) and commercial assay kit (Tridelta Development Ltd., Wicklow, Ireland) and cortisol was assayed using the Correlate-EIA kit from Assay Designs (Ann Arbour, MI, USA) according to the manufacturers’ instructions.

### Statistical analyses of the haematological, haptoglobin and cortisol data

Haematological, haptoglobin and cortisol data were tested for normality using PROC UNIVARIATE and the Shapiro-Wilk test and data that were not normally distributed were log transformed prior to statistical analyses. Haematological, physiological and rectal temperature data were analysed as repeated measures using the PROC MIXED procedure of SAS (Version 9.1, SAS Institute, Cary, NC). The first sample (d −4; sample 1) was used as the baseline covariate in the statistical analysis of the data. Animal was the experimental unit and was specified as a repeated measures effect, and the dependence within animal was modelled using an unstructured covariance structure. Differences between treatments were determined using the Tukey-Kramer test for multiple comparisons. Least squares means (Lsmeans) were considered significantly different at the *P* < 0.05 probability level.

### Leukocyte isolation from whole blood and subsequent RNA extraction

Thirty-six mL of blood collected in ACD tubes was pooled from each animal within 30 min of collection and split into three 12 mL aliquots. Red blood cells were lysed and the total leukocyte portion was collected and stored at −80 °C as described by O’Loughlin et al. [10]. A modified TRI Reagent extraction method was used to extract total RNA from leukocytes via homogenization of the pellet in TRI Reagent and the subsequent addition of chloroform followed by precipitation using isopropanol and ethanol. RNA was quantified using a Nanodrop Spectrophotometer (NanoDrop Technologies, Wilmington, DE, USA). RNA quality was assessed on an Agilent 2100 Bioanalyser (Agilent Technologies Ireland Ltd., Dublin, Ireland) and all RNA samples were found to have an RNA Integrity Number (RIN) greater than 9.0.

### RNA-seq library preparation

Single-read, 36 bp libraries were prepared for 6 animals per treatment, selected at random, on d 0, 1, 2 and 7 of the study. All 48 libraries were prepared from a starting material of 10 μg of high quality total RNA. Briefly, total RNA was heated at 65 °C for 5 min to disrupt secondary structures and the mRNA was isolated using 100 μL of Dyna oligo(dT) beads. This step was repeated in order to ensure the removal of genomic DNA, ribosomal RNA and other contaminating RNA. Fragmentation Reagent (Ambion) was used to fragment mRNA prior to synthesis of double-stranded cDNA. cDNA was synthesised using random hexamer primers and SuperScript II reverse transcriptase. Following second strand cDNA synthesis, each sample was purified using a QIAquick PCR spin column. Fragments were end repaired with T4 DNA polymerase and *E. coli* DNA Polymerase I Klenow fragment to remove 3’-overhangs and fill in 5’-overhangs. In order to prepare the cDNA fragments for adapter ligation, an additional “A” base was added to the 3’ end of the phosphorylated DNA fragments using Klenow Fragment 3’-to-5’ exo-. Adapters were ligated to the DNA fragments using Quick T4 DNA Ligase at room temperature for 15 min. Following adapter ligation, 10 μL of sample was loaded onto a 2% agarose gel in 1X TAE buffer. The gel was run at 120 V for 70 min. The gel was then visualised on a Dark Reader Transilluminator and a band of gel corresponding to the 250 bp ladder band was excised using a GeneCatcher Disposable Gel Excision tip. The DNA was then purified from this gel band using the QIAquick Gel Extraction kit and eluted in 30 μL of EB solution. PCR reactions were performed on the gel purified cDNA in a 50 μL reaction using Illumina supplied PCR primers 1.1 and 2.1 and Phusion High-Fidelity DNA polymerase for 15 cycles. Following the PCR step, the final libraries were purified using a QIAquick PCR spin column, quantified with a Qubit Fluorometer and stored at −80 °C until sequencing. Library size was confirmed by running 5 μL of each library on a gel and visually inspecting for the sole presence of a 250 bp band. Each library was sequenced in an individual lane on one of eight flowcells using a 36 bp single-end sequencing kit (version 4) at a concentration of 8.5 pM. Sequencing was carried out using an Illumina Genome Analyzer II at the Conway Institute, University College Dublin (Dublin, Ireland).

### Alignment of sequencing reads to the bovine genome and identification of differentially expressed genes

**Figure 2:**
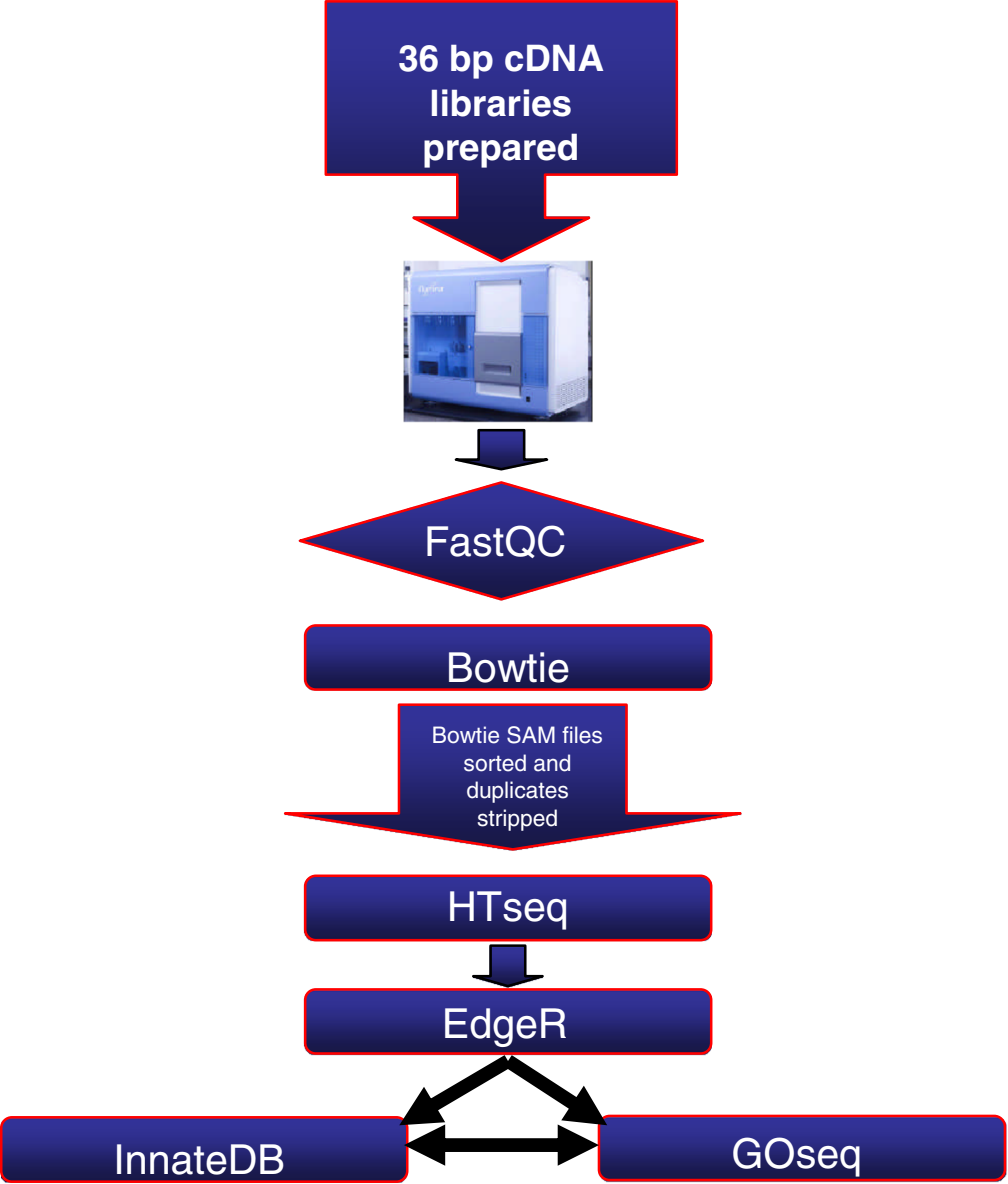
**Bioinformatic pipeline employed in the analysis of RNA-seq data.** Following sequencing on the GA_II_, FastQC was used to assess sequencing quality prior to aligning reads to the bovine genome with Bowtie. Counts were then assigned to each gene/exon with HTseq prior to the calculation of differential gene expression with EdgeR.

FastQC (version 0.9.1)(http://www.bioinformatics.bbsrc.ac.uk/projects/fastqc/) was used to assess the quality of reads from each lane of each flowcell based on a number of variables including per base sequence quality, per sequence quality score, per base N count and over-represented sequences (Figure 2). Bowtie (version 0.12.7) [23], the ultrafast, memory-efficient short read aligner was used to align the fastq output reads from the Genome Analyzer to the bovine genome (Btau4.0). The Bowtie script used allowed for no mismatches between a read and the reference genome and any read that had more than one reportable alignment to the reference genome was suppressed to avoid ambiguity in location. All retained reads were output in SAM format for further analysis. Using Strip_Sam_Duplicates, a script developed in-house (Dr. Chris Creevey, personal communication), multiple reads that aligned to the exact same position in the genome were removed due to their potential PCR bias introduced during library preparation. A single read with the highest PHRED quality score was retained at each of these locations. HTseq (v0.4.6p2)(http://www-huber.embl.de/users/anders/HTSeq) was used to convert aligned reads into counts per gene using the union model and the Ensembl v61 annotation of the bovine genome (ftp://ftp.ensembl.org/pub/release-61/fasta/bos_taurus/dna/). The above analysis used the number of reads mapping over the entire gene length to identify differentially expressed genes. This analysis could potentially miss cases where alternative splice variants are differentially expressed between comparisons. To investigate for such cases, reads were also mapped to exons using HTseq in order to identify differentially expressed exons instead of genes. Additional file 1: Table S1 contains the raw read counts broken down by gene and sample.

The R (version 2.11.0) Bioconductor package EdgeR (version 1.6.12) [24] was used to identify differentially expressed genes (and separately, exons) (Figure 2). EdgeR models data as a negative binomial distribution to account for biological and technical variation using a generalisation of the Poisson distribution model. Prior to assessing differential expression, data were first normalised across libraries using the trimmed mean of M-values normalization method [25]. Genes and exons were considered differentially expressed with a Benjamini-Hochberg false discovery rate (FDR)-corrected P-value < 0.05 and a fold change ≥ 2 [26]. All sequence data produced in this study has been submitted to NCBI GEO under accession number GSE37447.

### Over-representation analysis to identify gene ontology terms and pathways differentially regulated by weaning stress

Differential expression in RNA-seq data was calculated based on the number of reads aligning to a gene, thus there was greater statistical power to detect longer genes as significantly differentially expressed than shorter genes [27]. If a set of genes associated with Gene Ontology (GO) categories [28] are a non-random set with a preponderance of long or short genes, as is often the case, then transcript length will result in a bias of the GO analysis [29]. Therefore, it was necessary to correct for this bias in the RNA-seq data when identifying over-represented GO terms as there was a greater statistical power to detect GO categories with a prevalence of longer genes as over-represented.

Differentially expressed genes (DEG) from this study were analysed using the R package GOseq (version 1.1.7) which corrected for gene length bias [29]. The software package quantified the likelihood of a gene being differentially expressed as a function of transcript length which was incorporated into the final statistical test. GOseq was used to identify GO terms which were significantly more represented than expected by chance. GO terms were considered statistically significant at an FDR < 0.1.

The InnateDB pathway analysis tool [30] was used to identify significantly over-represented biological pathways using the hypergeometric test. Due to the minimal amount of pathway annotation available for the bovine genome, bovine genes and exons were converted to their human Ensembl orthologs prior to analysis ( Additional file 2: Table S2). Pathways were considered significantly over-represented with an FDR < 0.1. A potential concern was that the pathway analysis of RNA-seq data may be subject to the same gene length related biases affecting the GO analysis. To determine whether this was the case, GOseq was also used (using imported InnateDB pathway annotations, March 15, 2011 release) to identify over-represented pathways. The same pathways were identified by InnateDB and GOseq and as such InnateDB (an interactive web-based platform) was utilised in the study.

### oPOSSUM analysis to detect transcription factors likely to contribute to differential gene expression

A further analysis was performed using the significantly differentially expressed genes detected by EdgeR in order to identify transcription factors involved in gene expression changes during the stress response. Significantly differentially expressed human orthologs from each comparison were divided into two groups for analysis, up-regulated and down-regulated genes. These groups of genes were then analysed using the publicly available web-based system, oPOSSUM [31] to detect over-represented transcription factor binding sites (TFBS) in the promoter regions of each set of genes. To detect common TFBS, the approach was taken to examine 5,000 base pairs upstream and downstream of each transcription start site (TSS). Transcription factors were matched based on their corresponding binding site and over-represented transcription factors were considered to be significant at a Z-score ≥ 10 and a Fisher score ≤ 0.01.

## Results

### Physiological response to weaning stress at housing on neutrophils, lymphocytes, platelets, cortisol and haptoglobin

**Figure 3:**
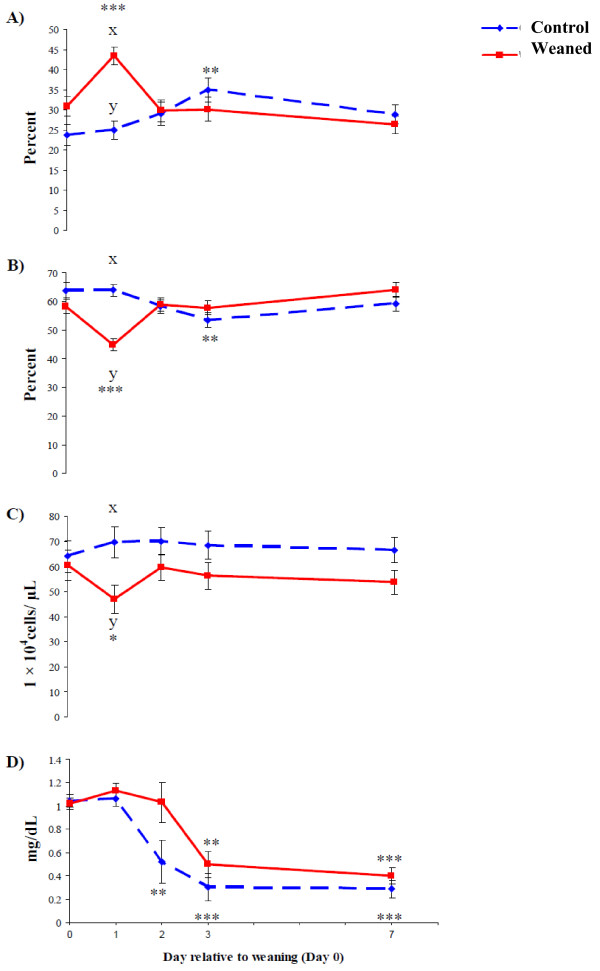
**Physiological profiles of weaned and control calves in response to stress.** Neutrophil percentage (**A**) rapidly increased on day 1 following weaning, indicating a profound stress response that was reciprocally matched by lymphocyte percentage (**B**). Platelet number (**C**) also decreased in response to weaning while haptoglobin (**D**) decreased in both weaned and control calves. * = *P* < 0.05, ** = *P* < 0.01, *** = *P* < 0.001 Lsmeans differ relative to baseline (Day 0). ^x,y^Lsmeans differ between location by *P* < 0.05.

There was a treatment × sampling time (*P* < 0.001) interaction for total leukocyte number on d 3 where leukocyte number was greater in control calves compared with baseline with no change in weaned calves relative to baseline (Table 1). There was a treatment ×sampling time interaction (*P* < 0.001) for neutrophil percentage whereby on d 1 it increased in weaned calves before returning to pre-weaning baseline, whereas control calves differed from baseline on d 3 (Figure 3a). This resulted in a higher neutrophil percentage in weaned calves compared with control calves on day 1. There was a treatment × sampling time interaction (*P* < 0.001) for lymphocyte percentage, which was converse to that of neutrophils (Figure 3b). This relationship was reflected in the N:L ratio, which increased from pre-weaning baseline in weaned calves on d 1 and was higher than control calves at this time, while it also increased from baseline in weaned calves on d 3. Platelet number decreased from baseline on d 1 in weaned calves and was also lower than control calves at this sampling time-point (Figure 3c). There was a treatment × sampling time interaction for monocyte percentage (*P* < 0.05) and MCHC (*P* < 0.05) where monocytes were lower than baseline in weaned calves on d 7 and MCHC increased from baseline on d 1 in weaned and control calves, and on d 3 in weaned calves.

**Table 1:** **Effect of stress on haematological variables**^**1**^**in control and weaned calves**

		**Days post weaning**					***P*-values**		
**Variable**		0	1	2	3	7	T	S	T × S
**Total Leukocytes**	C	13.3 ± 0.97	13.8 ± 1.44	13.9 ± 0.71	15.6^bx^ ± 1.08	13.9^x^ ± 0.61	NS	NS	*
(×10^3^ cells/μL)	W	12.2 ± 0.91	13.2 ± 1.34	12.1 ± 0.66	12.2^y^ ± 1.01	11.3^y^ ± 0.57			
**Neutrophils, %**	C	23.7 ± 2.63	24.9^x^ ± 2.30	28.9 ± 2.92	34.9^b^ ± 3.12	28.9 ± 2.29	NS	NS	***
	W	30.9 ± 2.45	43.4^cy^ ± 2.14	29.7 ± 2.72	30.1 ± 2.90	26.3 ± 2.14			
**Lymphocytes, %**	C	63.7 ± 2.87	63.9^x^ ± 2.17	58.3 ± 2.44	53.3^b^ ± 2.59	59.0 ± 2.50	NS	NS	***
	W	58.4 ± 2.67	44.9^cy^ ± 2.02	58.8 ± 2.27	57.8 ± 2.41	64.1 ± 2.33			
**N:L Ratio**	C	0.38 ± 0.076	0.41^x^ ± 0.061	0.52 ± 0.079	0.69^b^ ± 0.098	0.51 ± 0.056	NS	NS	***
	W	0.57 ± 0.071	0.99^cy^ ± 0.057	0.52 ± 0.074	0.54 ± 0.091	0.42 ± 0.052			
**Eosinophils, %**	C	2.7 ± 0.46	1.9^a^ ± 0.23	3.4 ± 0.86	3.7 ± 0.89	3.5 ± 1.18	NS	NS	NS
	W	1.6 ± 0.43	1.9 ± 0.21	2.3 ± 0.79	1.9 ± 0.83	1.8 ± 1.09			
**Monocytes, %**	C	6.6 ± 0.36	7.1 ± 0.44	7.2 ± 0.39	6.1 ± 0.63	5.9 ± 0.45	NS	NS	*
	W	7.5 ± 0.33	6.9 ± 0.41	6.9 ± 0.36	7.5 ± 0.59	5.7^b^ ± 0.42			
**RBC**	C	11.9 ± 0.21	11.8 ± 0.19	12.3^b^ ± 0.24	12.1 ± 0.27	12.1 ± 0.28	NS	NS	NS
(×10^6^ cells/μL)	W	11.6 ± 0.22	11.6 ± 0.21	11.8 ± 0.26	11.8 ± 0.29	11.8 ± 0.29			
**HCT, %**	C	34.6 ± 0.68	34.4 ± 0.69	35.9^b^ ± 0.84	35.5 ± 0.85	35.3 ± 0.81	NS	*	NS
	W	33.5 ± 0.64	33.4 ± 0.65	34.1 ± 0.78	34.5 ± 0.79	34.2 ± 0.76			
**HGB** (g/dL)	C	13.0 ± 0.19	13.1 ± 0.25	13.5^b^ ± 0.28	13.4 ± 0.29	13.4 ± 0.29	NS	*	NS
	W	12.7 ± 0.19	12.8 ± 0.23	12.9 ± 0.26	12.9 ± 0.28	12.9 ± 0.27			
**MCHC** (g/dL)	C	37.7 ± 0.28	38.1^b^ ± 0.24	37.7 ± 0.22	37.7 ± 0.23	37.9 ± 0.29	NS	NS	*
	W	37.9 ± 0.26	38.5^c^ ± 0.22	37.8 ±0.21	37.5^a^ ± 0.21	37.7 ± 0.27			
**Platelet number**	C	640.1 ± 63.06	697.2^x^ ± 60.73	700.4 ± 55.59	684.8 ± 57.03	665.2 ± 49.91	NS	*	NS
(×10^3^ cells/μL)	W	604.5 ± 58.79	469.4^ay^ ± 56.62	596.4 ± 51.83	562.3 ± 53.17	536.3 ± 46.54			

^**1**^Haematological variables; total leukocyte, neutrophil and lymphocyte percentage, neutrophil:lymphocyte (N:L) ratio, eosinophil and monocyte percentage, red blood cell (RBC) number, haematocrit (HCT) percentage, haemoglobin (HGB) and mean corpuscular haemoglobin concentration (MCHC). The values are expressed as least squares means (Lsmeans) ± s.e.

C = Control, W = Weaned, T = treatment, S = sampling time, T × S = treatment × sampling time interaction, NS = not significant (*P* > 0.05).

* = *P* < 0.05, ** = *P* < 0.01, *** = *P* < 0.001.

^a,b,c^Within rows, Lsmeans differ from pre-weaning baseline by *P* < 0.05, *P* < 0.01 and *P* < 0.001, respectively.

^x,y^Between rows, Lsmeans differ between treatments by *P* < 0.05.

There was a treatment × sampling time interaction (*P* < 0.05) for serum cortisol concentration whereby on d 1 concentration was greater in weaned calves compared with control calves but not subsequently (*P* > 0.05) (Table 2). Plasma concentration of haptoglobin decreased (*P* < 0.001) from baseline on d 2 in control calves and on d 3 in weaned calves and subsequently remained lower (*P* < 0.001) than baseline (Figure 3d).

**Table 2:** Effect of stress on circulating cortisol and haptoglobin concentrations in control and weaned calves

		**Days post weaning**					***P*-values**		
**Variable**		0	1	2	3	7	T	S	T × S
**Cortisol**	C	6.1 ± 1.17	4.3^x^ ± 1.36	8.5 ± 1.81	5.7 ± 1.31	9.7 ± 2.29	NS	*	*
(ng/mL)	W	6.3 ± 1.09	8.4^y^ ± 1.27	8.4 ± 1.68	8.4 ± 1.23	6.5 ± 2.14			
**Haptoglobin**	C	1.04 ± 0.052	1.07 ± 0.068	0.52^b^ ± 0.184	0.30^c^ ± 0.122	0.29^c^ ± 0.076	NS	***	NS
(mg/mL)	W	1.02 ± 0.049	1.14 ± 0.063	1.03 ± 0.173	0.49^b^ ± 0.114	0.39^c^ ± 0.071			

The values are expressed as least squares means (Lsmeans) ± s.e.

C = Control, W = Weaned, T = treatment, S = sampling time, T × S = treatment × sampling time interaction, NS = not significant (*P* > 0.05).

* = *P* < 0.05, ** = *P* < 0.01, *** = *P* < 0.001.

^a,b,c^Within rows, Lsmeans differ from pre-weaning baseline by *P* < 0.05, *P* < 0.01 and *P* < 0.001, respectively.

### Preliminary analysis of RNA-seq data

Forty-eight libraries representing 12 animals in two different treatments (control (n = 6) and weaned (n = 6)) on d 0, 1, 2 and 7 were prepared from total leukocyte mRNA. Collectively, this resulted in 1,089,256,422 sequenced reads with an average of 22,692,824 reads per lane ( Additional file 3: Table S3). On average, 4,479,044 reads per lane failed to align, while a further 4,325,899 aligned to multiple genomic locations. A conservative approach was adapted towards identical reads aligning to the same genomic position whereby all but one of these reads was removed at each genomic location. An average of 7,402,570 reads per lane were removed due to their potential role in introducing PCR bias, leaving 6,485,330 unique reads per lane, or approximately 28% of reads, successfully aligned to a single genomic location. These were used for subsequent analysis, comparable to other recent RNA-seq studies [32,33].

### Pathway analysis of differentially expressed genes to characterise the transcriptomic differences between weaned and control calves

**Figure 4:**
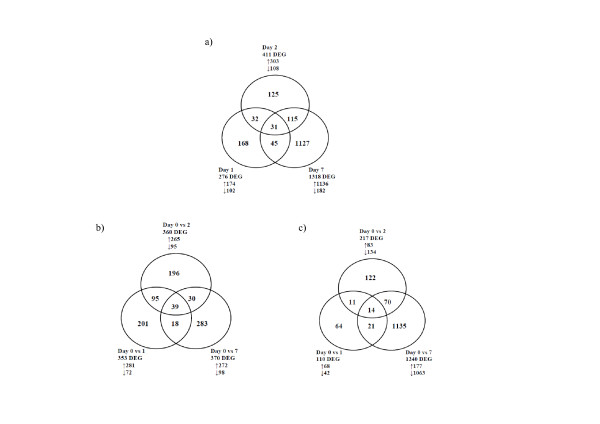
**Venn diagram analysis of the number of differentially expressed genes between treatments.** The comparison of weaned versus control calves at each time point is shown in (**a**) while (**b**) represents the comparison of differentially expressed genes from day 1, 2 and 7 to d 0 in weaned calves (response to weaning stress) and (**c**) shows the comparison of day 1, 2 and 7 to d 0 in control calves (response to housing stress).

In order to identify key differences between the weaned and control treatments, InnateDB pathway analysis was utilised and identified four main pathway categories, cytokine signalling, transmembrane transport, haemostasis and G-protein-coupled receptor (GPRC) signalling as statistically over-represented among differentially expressed genes in the weaned animals in comparison with controls ( Additional file 4: Table S4; Additional file 5: Table S5). Where a pathway was not significantly over-represented, no genes are listed. Graphic representations of the number of differentially expressed bovine genes are represented in Figure 4 ( Additional file 2: Table S2 contains full list of DEG).

### Cytokine signalling

On d 1, the cytokine signalling pathway was transcriptionally activated between treatments with nine genes in this pathway up-regulated in weaned calves. However, this pathway was not statistically over-represented on d 2 and d 7, despite the differential expression of a number of cytokines at these other time points.

### Transmembrane transport

Similar to the cytokine signalling pathway, the transmembrane transport pathway was transcriptionally activated on d 1 with a number of differences observed between weaned and control calves. There was an up-regulation of six genes in weaned calves, all of which are members of the solute-carrier gene superfamily. Of the nine differentially expressed transmembrane transport pathway genes, eight were up-regulated on d 2. By d 7, this pathway was no longer significantly over-represented.

### Haemostasis

Two genes, phosphodiesterase 5A, cGMP-specific (PDE5A) and Inositol 1,4,5-triphosphate receptor-associated cGMP kinase substrate (MRVI1), were altered in the haemostatic pathway on d 1 with both up-regulated in weaned animals. This changed on d 2 with two genes, collagen type 1 alpha 1 (COL1A1) and collagen type 1 alpha 2 (COL1A2) down-regulated in weaned calves. These two genes were down-regulated on d 7, along with the up-regulation of a third gene, the surface integrin α2β1.

### GPCR signalling

While the GPCR signalling pathway was not significantly over-represented on d 1, five genes were identified as playing a role in this pathway on d 2. Expression of both the beta-1 and −3 adrenoreceptors, responsible for activation of adenylate cyclase activity, was suppressed in weaned calves, while the 5-hydroxytryptamine (serotonin) receptor 1B (HTR1B) was up-regulated. However, on d 7, a different set of genes known as regulators of G-protein signalling were differentially expressed between treatments with five of the six genes up-regulated in weaned calves.

### Gene ontology analysis of differentially expressed genes to characterise the transcriptomic differences between weaned and control calves

**Figure 5:**
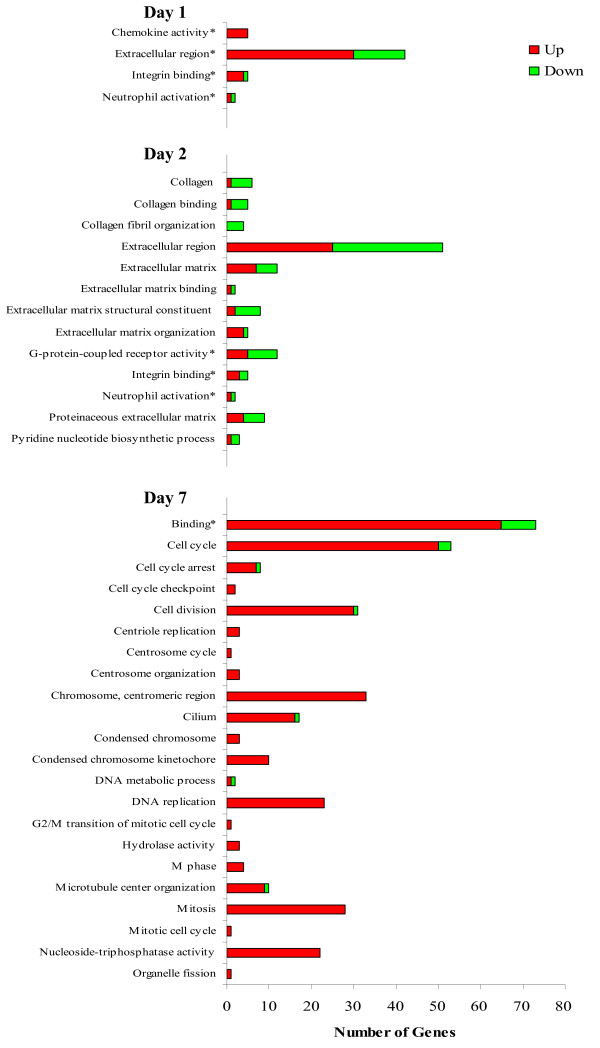
**Significant GO terms returned by GOseq analysis of genes differentially expressed between weaned and control calves.** On day 2, DEG were primarily assigned to GO terms involved in extracellular matrix structure and function, particularly in the secretion of hormones, cytokines and other ligands. However, by day 7, almost of DEG identified belonged to GO terms involved in mitotic cell division. *0.2 < FDR > 0.1.

GOseq analysis did not identify any significant GO terms between weaned and control calves on d 1, although over 50 genes fell into the GO terms "chemokine activity", "integrin binding", "neutrophil activation", and "extracellular region" (Figure 5).

Thirteen enriched GO terms were returned for the differentially expressed genes on d 2, principally relating to the extracellular region and its components. Of the eight differentially expressed genes annotated to "extracellular matrix structural constituent", six were down-regulated in weaned animals, including the collagen genes collagen type I, III, IV and V. The GO term "extracellular region" encompassed the largest group of DEG on d 2 with fifty-one differentially expressed genes between weaned and control calves. This GO term was evenly split with twenty-five up-regulated and twenty-six down-regulated in weaned animals. Up-regulated genes included the platelet-derived growth factor C (PDGFC) and the lymphocyte apoptotic inhibitor, CD5L, among a range of other genes involved in extracellular matrix structure and function, particularly in the secretion of hormones, cytokines and other ligands. On d 7, 22 over-enriched GO terms, predominantly involved in mitotic cell division, were identified.

### Pathway analysis of differentially expressed genes to characterise the transcriptomic response of bovine leukocytes to weaning stress at housing days 1, 2 and 7 after weaning

This analysis examined the response to weaning at housing by comparing gene expression on d 1, 2 and 7 with the pre-weaning baseline on d 0 (Figure 4).

### Cytokine signalling

On d 1, fourteen differentially expressed genes were annotated to the cytokine signalling pathway, with ten up-regulated following weaning including the neutrophil and lymphocyte chemoattractants CXCL5, CXCL7, CXCL8, CCL2, CCL24 and XCL2 ( Additional file 6: Table S6). This pathway remained significantly activated on d 2 post-weaning with twelve of the sixteen differentially expressed genes up-regulated. While cytokines, chemokines and their receptors accounted for all the differentially expressed genes in this pathway on d 1, by d 2, there were fewer chemokines induced, although a number of inflammatory mediators, including IL-1, were up-regulated. All four of the genes assigned to the cytokine signalling pathway on d 7 (IFN-γ, JAK2, LRPPRC, LIF) were up-regulated and represent a shift in the cytokine signalling network from chemotaxis towards mediated inflammatory response.

### Transmembrane transport

A number of differentially expressed genes were involved in transmembrane transport, with seven of the eight increasing in response to weaning on d 1. These included two each of the glucose carrier family SLC5 and the potassium dependent sodium/calcium exchanger SLC24 family, in addition to the neurotransmitter transporter, SLC6A15. On d 2, transmembrane transport remained similarly expressed to d 1, with members of the SLC5, SLC6 and SLC24 families being the most prominent of the 10 up-regulated genes in this pathway. By d 7, the transmembrane transport pathway was no longer significantly over-represented, although a number of genes involved in transport were still differentially expressed.

### Haemostasis

Of the ten genes differentially expressed in the haemostatic pathway on d 1, eight were up-regulated from baseline expression levels, principally involved in inflammation and the adhesion of platelets while type I collagen (COL1A1 and COL1A2) was down-regulated. The down-regulation of these two genes was also found on d 2 and 7. However, the majority of the 13 genes involved in haemostasis on d 2 were up-regulated, being mostly involved in the adhesion of platelets to exposed collagen and the subsequent process of coagulation and immune cell activation. A shift in the haemostatic pathway occurred on d 7 with five of the seven differentially expressed genes in this pathway down-regulated from the pre-weaning baseline, although the α2β1 integrin remained up-regulated.

### GPCR signalling

Only two genes in the GPCR pathway, the multifunctional vasodilator adrenomedullin (ADM) and the ADM receptor, calcitonin receptor-like receptor (CALCRL), were altered on d 1 with a down-regulation of ADM accompanied by an up-regulation of CALCRL. However, on d 2 twenty-four of the thirty-three differentially expressed genes in the GPCR signalling pathway were up-regulated. These included significant numbers of both G-protein-coupled receptors and their diverse ligands, in addition to RGS genes. Despite a reduction of signalling in the GPCR pathway on d 7, seventeen genes were still altered with eleven having increased expression levels including cytokines, neuropeptides, GPCR and two members of the RGS family.

### Gene ontology analysis of differentially expressed genes to characterise the transcriptomic response of bovine leukocytes to weaning stress at housing 1, 2 and 7 days after weaning

**Figure 6:**
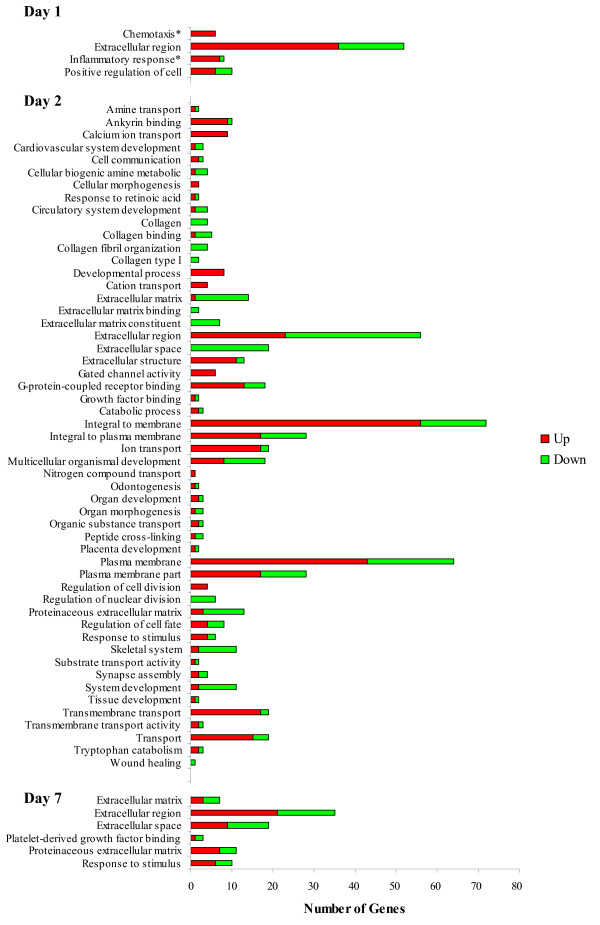
**Significant GO terms returned by GOseq analysis of genes differentially expressed in response to weaning stress.** A large number of GO terms were identified in response to weaning, predominantly involved in the extracellular region and plasma membrane. This indicates that a large number of genes were involved in cell signalling and includes a number of cytokines. The presence of the "extracellular region", "extracellular space", "extracellular matrix" and "proteinaceous extracellular matrix" on day 7 is similar to day 2 but suggests that the magnitude of the day 2 response had reduced. *0.2 < FDR > 0.1.

GOseq returned a single significant GO term on d 1 (Figure 6). A total of fifty-two genes were annotated to the GO term "Extracellular region", the majority of which (n = 36) were up-regulated following weaning. This term covers a broad group of genes primarily involved in cell signalling and includes a number of cytokines. Eighty-seven significant GO terms were returned as over-enriched on d 2. The two largest GO terms from d 2, "plasma membrane" and "integral to membrane" reveal that a vast majority of the annotated genes were involved in cell signalling, adhesion and ion transport. "Cell communication" and a number of GO terms associated with transmembrane transport similarly indicate the role of cell signalling. This had greatly reduced by d 7, where only six GO terms were associated with differentially expressed genes on d 7. The presence of the "extracellular region", "extracellular space", "extracellular matrix" and "proteinaceous extracellular matrix" indicate a role of a number of genes in cell signalling and interactions. "Extracellular region" covered 35 genes with 60% of these up-regulated following weaning.

### Pathway analysis of differentially expressed genes to characterise the transcriptomic response of bovine leukocytes to housing stress 1, 2 and 7 days after housing

**Figure 7:**
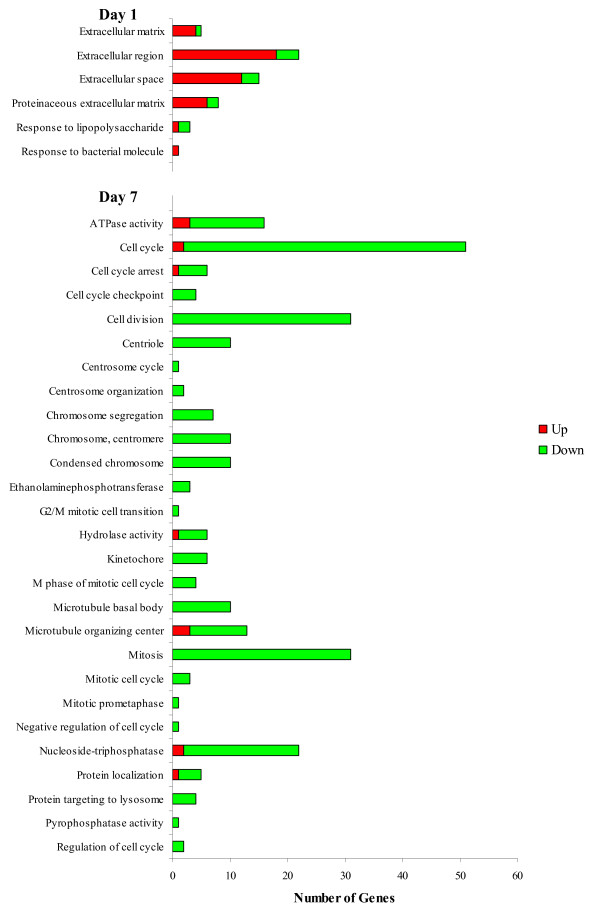
**Significant GO terms returned by GOseq analysis of genes differentially expressed in response to housing stress.** A similar response to weaned calves occurred in control calves on day 1 with an up-regulation of genes involved in the extracellular region. However, by day 7, there was a large down-regulation of genes in control calves, predominantly involved in the cell cycle. This is responsible for the large difference identified between treatments on day 7.

A profile similar to weaned calves existed for control calves throughout the study, albeit the magnitude of expression was significantly lower (Figure 4; Additional file 7: Table S7). The GO data are represented in Figure 7.

### Analysis of exons to identify potential alternative splicing events

Although power to detect alternative splicing is limited with single-end reads, we identified a number of genes which, when counting reads over the entire gene were not detected as differentially expressed, but which had at least one differentially expressed exon (Table 3). Genes with differentially expressed exons ( Additional file 8: Table S8) were mapped to their human orthologs and pathway analysis was carried out to identify whether any biological processes were statistically over-represented among these genes.

**Table 3:** Differentially expressed exons and estimates of alternate splicing

	**Number of differentially expressed genes**	**Number of differentially expressed exons**	**Number of genes with differentially expressed exons**	Estimated number of Genes with alternate splicing*
**Weaned versus Control**				
Day 1	276	48	28	8
Day 2	411	97	56	19
Day 7	1318	2091	1217	689
**Weaning response**				
Day 0 vs 1	353	227	190	160
Day 0 vs 2	360	104	58	22
Day 0 vs 7	370	36	25	2
**Housing response (Control)**				
Day 0 vs 1	110	0	0	0
Day 0 vs 2	217	32	22	9
Day 0 vs 7	1240	3921	1734	1087

*Non differentially expressed genes with differentially expressed exons.

Ten genes were alternatively spliced between weaned and control calves on d 1. On d 2, three genes involved in metabolism were alternatively spliced ( Additional file 9: Table S9). The majority of alternative splicing was observed on d 7 with analysis revealing seventy-two over-represented pathways including mitotic cell cycle, metabolism of RNA, androgen receptor signalling, RNA transport and T cell receptor signalling.

A number of alternative splicing events occurred in weaned calves relative to pre-weaning with the most significant alterations occurring on d 1 ( Additional file 9: Table S9). Some of the most biologically and statistically significant pathways were toll receptor cascades, innate immune signalling, CD4 T cell receptor signalling and the phagosome. However, on d 2, transmembrane transport was the only over-represented term from the alternative splicing data.

While no alternative splicing events were identified relative to the pre-housing baseline in the control calves, nine genes were identified on d 2, predominantly involved in Wnt signalling ( Additional file 9: Table S9). Pathway analysis of alternatively spliced genes on d 7 was similar to the profile of differentially expressed genes on d 7 with a number of pathways involved in cell cycle identified.

### Identification of transcription factors involved in the bovine stress response based on common transcription factor binding sites of differentially expressed genes

Transcription factor analysis with oPOSSUM revealed a number of transcription factors to be responsible for differential gene expression between weaned and control calves ( Additional file 10: Table S10) with a similar pattern for weaned calves compared to the pre-weaning baseline ( Additional file 11: Table S11). The transcription factor analysis of differentially expressed genes from control calves versus the pre-housing baseline revealed no over-represented transcription factor binding sites in the differentially expressed gene subsets on d 1, although significant TFBS were identified on d 2 and d 7 ( Additional file 12: Table S12).

## Discussion

Weaning is a multifactorial stressor, often encompassing separation from the dam and peers, mixing with unfamiliar animals, housing, novel handling and a change in diet and environment. The results of the present study demonstrate that the leukocyte transcriptomic environment is profoundly altered by weaning stress, at least up to 7 days post-weaning and that a number of processes are transcriptionally activated to increase immune cell adhesion and migration, potentially serving to increase immune surveillance following exposure to weaning stress.

Neutrophilia resulting from a stressor, as seen in this study, has been frequently reported in both cattle [5,6,9,10,19] and humans [34]. This can partly be attributed to a surge of cortisol [35,36] which may result in a series of physiological alterations to neutrophil function including the release of a large number of immature neutrophils from the bone marrow [37,38], an increase in neutrophil chemotaxis [39] and a reduction in the neutrophil apoptotic rate [35,36,40,41]. However, circulating cortisol concentrations peak early in the stress response and it is probable that the infrequent sampling intervals employed in the current study likely missed the true peak [42,43].

It has previously been reported that stress-induced cortisol surges inhibit CD62L (L-selectin) expression on bovine neutrophils, thus reducing endothelial adhesion and subsequent migration to sites of infection [19,44,45]. However, several other bovine transport [41], castration [46] and weaning [10] studies have failed to identify this suppression transcriptionally. Furthermore, L-selectin expression may not be necessary to maintain leukocyte rolling as platelets have been reported to re-establish leukocyte trafficking in CD62L deficient mice [47]. It is clear that a number of genes involved in integrin binding are up-regulated in weaned calves and that, while no difference in the expression of CD62L was identified, several other important integrins involved in cell adhesion and migration were up-regulated, in addition to a clear activation of platelets. The induction of a number of genes involved in transmembrane transport, including the glucose carrier family SLC5, the potassium dependent sodium/calcium exchanger SLC24, and the sodium and chloride dependent neurotransmitter transporter, SLC6A15, demonstrates an increased role for transmembrane transport of small molecules to leukocytes [48], and further work is necessary to examine if this may be involved in their functional activation.

Cytokines play an important role in the immune response, primarily functioning as inflammatory mediators of the innate immune system [49]. The transcriptional induction of genes involved in chemotaxis provides a clear mechanism by which leukocyte function was altered. Chemokines are primarily involved in the recruitment of immune cells to inflammatory sites [50,51], and their increased expression indicates that weaning stress results in transcriptional alterations that may enhance leukocyte recruitment and activation. Despite a preponderance of differentially expressed cytokine transcripts known to interact directly with neutrophils [52-54], it is likely many of these cytokines also influenced a number of other cell populations including lymphocytes, monocytes and platelets [55-58]. This is perhaps best demonstrated by the large induction of the GPCR signalling pathway with a number of regulators of G-protein signalling (RGS) genes remaining up-regulated throughout the course of this study, suggesting the rate of GPCR signalling was increased [59]. Transcriptional signatures suggest neutrophils, monocytes and lymphocytes may have had an enhanced capability to exit the vasculature and increase immune surveillance through an up-regulation of a number of genes responsible for chemotaxis and integrin binding, indicating enhanced leukocyte function in response to stress, However, it would be of interest in future studies to validate this hypothesis via cell adhesion and migration assays in order to confirm the corresponding physiological state.

Inflammation is tightly linked to haemostasis, an important innate immune defence mechanism primarily intended to arrest the bleeding process [60], although it has recently been shown that platelets have additional functionality and interact with cells of the innate immune system via secretion of cytokines, chemokines and other inflammatory mediators [61-64]. In the current study, pathway analysis suggests that the process of haemostasis was activated as a result of weaning, and based on this activation, may have resulted in increased platelet aggregation, reducing the number of platelets freely available in circulation, as identified in weaned calves on day 1. A number of genes involved in collagen synthesis were differentially expressed with the overwhelming majority down-regulated in weaned animals, both in relation to control calves and the pre-weaning baseline. An early reaction to tissue damage is the release of collagen into blood vessels where it acts as a potent agonist of platelets and other leukocytes [65]. This is mediated by cytokine induced collagenase secretion along with the down-regulation of collagen mRNA by cortisol [66-68], aiding in the rapid migration of leukocytes through the extracellular matrix (ECM) to sites of inflammation [69]. An examination of genes involved in the extracellular region revealed the presence of a number of genes responsible for the activation and coagulation of platelets [70-72], in addition to platelet derived chemokines, such as CCL2 [55], CXCL5 [62], CXCL7 [73] and platelet-derived growth factor C (PDGFC) [74] which may have facilitated the recruitment of neutrophils, monocytes, lymphocytes and other leukocytes to susceptible tissues, signifying that stress can transcriptionally activate an acute inflammatory response through interactions with platelets and the haemostatic system.

A large number of genes were differentially expressed on d 7 in weaned animals when compared with baseline expression values. While we have previously reported a detectable signature of inflammatory candidate genes in bovine leukocytes 7 days following weaning [10], it was unexpected that the expression of such a large number of genes (>300) would remain altered for so long following weaning, although it has been previously reported that a number of physiological parameters, including plasma fibrinogen and haptoglobin concentrations, and total leukocyte number had not returned to baseline in weaned calves 35 days following weaning [19]. Results from the current study indicate that weaning can have a profound impact on the leukocyte transcriptome for at least 7 days post-weaning and suggests that the stress of weaning can have far reaching consequences for the homeostasis of the transcriptome. The majority of these genes were up-regulated and principally involved in cell signalling and function with a number of cytokines involved in the Th1 response [75]. An increase in the magnitude of the stressor can quickly result in deleterious effects [76] as a prolonged inflammatory response can cause severe tissue damage [41,77].

The greatest induction of genes in the control calves also occurred on d 7, a rather unexpected result given that these animals were not additionally exposed to any other stressor. However, the profile for these calves was much different than that of the weaned animals with the cytokine signalling, transmembrane transport, haemostasis and GPCR signalling pathways all having returned to baseline by d 7. Rather, almost 86% of the genes, which related to DNA replication and the cell cycle, were down-regulated on d 7 in relation to the pre-housing baseline. While it is clear that housing results in a molecular stress response on d 1 and 2, the results of d 7 require further examination as a similar effect was not found in the weaned treatment, despite these calves also being housed in addition to weaning. However, further research is required to assess the long term effect of housing stress in weaned calves. In the first 2 days following housing, a similar stress induced gene expression profile is present in both control and weaned calves, with a greater magnitude of induction in weaned calves exposed to the accumulative stress of housing, weaning and social reorganisation.

## Conclusions

A major strength of this study is that it represents the first application of RNA-Seq technology for genomic studies in bovine leukocytes in response to weaning stress. Weaning stress induced a significant number of transcriptional changes that are reflected in the physiological alterations identified in cellular distribution. The most important finding of this study was that simultaneously weaning and housing of calves produces a perturbation to the homeostasis of the leukocyte transcriptome which was still present 7 days following weaning. Weaning at housing induced activation of a number of cytokine, chemokine and integrin transcripts which may alter the immune system whereby the ability of a number of cells of the innate and adaptive immune systems to locate and destroy pathogens is enhanced. Weaning also appears to elicit an acute inflammatory response in calves. However, this may have potentially negative consequences for tissues that are at risk of damage due to inflammation. Additional research is required to align these two conclusions, although it is likely a case where an initial beneficial anti-pathogenic response gives way to damage due to inflammation over a number of days when the stressor is not eliminated. It is also clear that housing results in a less marked stress response than weaning, as identified by a reduced induction in gene expression following housing. Future studies should examine the processes that have been identified in this study as playing a critical role in immunocompetence of weaned calves and should principally address the ability of calves to mount an immune response to viral and bacterial challenge following weaning or exposure to other stressors. This study has identified regulatory gene networks that are stress activated, and may be modulated by glucocorticoids in leukocytes, and provides a mechanistic framework to characterise the multifaceted nature of weaning stress adaptation in beef calves.

## Competing interests

The author(s) declare that they have no competing interests.

## Authors’ contributions

BE and MMcG designed the study. AOL performed the experiments. AOL, DJL and BE analysed the data and AOL prepared the manuscript. AOL, DJL, MMcG, SD, MMcC and BE contributed to, read and approved the final manuscript.

## Supplementary Material

Additional file 1 **Table S1.**This summary file contains the raw read counts broken down by gene and sample. Click here for file

Additional file 2 **Table S2.**Differentially Expressed Genes. This file contains a list of the significantly differentially expressed (FDR < 0.05) genes at each comparison and their human orthologs, when available. Click here for file

Additional file 3 **Table S3.**Lane Assignment and Preliminary Analysis of RNA-seq Reads. This file contains a table containing the lane assignments for each sample along with a summary of reads by lane. Click here for file

Additional file 4 **Table S4.**Significantly differentially expressed pathways between weaned and control calves. Click here for file

Additional file 5 **Table S5.**Over-represented InnateDB pathways involved in the bovine stress response. This file contains a list of pathways identified with InnateDB as being over-represented among the differentially expressed genes for each comparison. Click here for file

Additional file 6 **Table S6.**Significantly differentially expressed pathways in weaned calves. Click here for file

Additional file 7 **Table S7.**Significantly differentially expressed pathways in control calves. Click here for file

Additional file 8 **Table S8.**Alternatively spliced genes. This file contains a list of genes that underwent alternative splicing for each comparison. Click here for file

Additional file 9 **Table S9.**Pathways affected by alternative splicing. This file contains a list of pathways identified as over-represented following analysis with alternatively spliced genes. Click here for file

Additional file 10 **Table S10.**Significantly over-represented transcription factors based on transcription factor binding sites of up- and down-regulated genes between weaned and control calves. This file contains a table of transcription factors identified as having a role in the regulation of genes differentially expressed between weaned and control animals using oPOSSUM. Click here for file

Additional file 11 **Table S11.**Significantly over-represented transcription factors based on transcription factor binding sites of up- and down-regulated genes following weaning. This file contains a table of transcription factors identified as having a role in the regulation of genes differentially expressed following weaning using oPOSSUM. Click here for file

Additional file 12 **Table S12.**Significantly over-represented transcription factors based on transcription factor binding sites of up- and down-regulated genes following housing. This file contains a table of transcription factors identified as having a role in the regulation of genes differentially expressed following housing stress using oPOSSUM. Click here for file
