# Supplementary material for: Transcriptomic analysis of the stress response to weaning at housing in bovine leukocytes using RNA-seq technology
Source: BMC Genomics. 2012 Jun 18;13:250. doi: 10.1186/1471-2164-13-250 (PMC3583219; doi:10.1186/1471-2164-13-250)
Supplement: Additional file 3 — Table S3.Lane Assignment and Preliminary Analysis of RNA-seq Reads. This file contains a table containing the lane assignments for each sample along with a summary of reads by lane. [file 1471-2164-13-250-S3.doc]

| **Supplementary Table 3. Lane assignments and preliminary analysis of RNA-seq reads** | | | | | | | | | |
| --- | --- | --- | --- | --- | --- | --- | --- | --- | --- |
| **Flowcell** | **Lane** | **Sample ID** | **Group** | **Raw reads** | **Failed to align** | **Aligned to multiple sites** | **Accepted alignments** | **Multiple Reads (PCR) removed** | **Retained Reads** |
| 100202 | 3 | 1755(4) | Con | 23807742 | 4234985 (17.79%) | 4364588 (18.33%) | 15208169 (63.88%) | 8139770 | 7068399 (29.69%) |
| 100202 | 5 | 1755(2) | Con | 24103370 | 3893758 (16.15%) | 4792467 (19.88%) | 15417145 (63.96%) | 6989093 | 8428052 (34.97%) |
| 100202 | 6 | 1755(3) | Con | 22900561 | 3721237 (16.25%) | 4298948 (18.77%) | 14880376 (64.98%) | 6124809 | 8755567 (38.23%) |
| 100222 | 1 | 1755(6) | Con | 25308344 | 4848638 (19.16%) | 4515039 (17.84%) | 15944667 (63.00%) | 10689018 | 5255649 (20.77%) |
| 100222 | 2 | 1758(2) | wean | 24731241 | 4972679 (20.11%) | 4434545 (17.93%) | 15324017 (61.96%) | 9606503 | 5717514 (23.12%) |
| 100222 | 3 | 1758(3) | Wean | 27200717 | 4667698 (17.16%) | 5216682 (19.18%) | 17316337 (63.66%) | 9274689 | 8041648 (29.56%) |
| 100222 | 4 | 1758(4) | Wean | 27491775 | 4418265 (16.07%) | 5246494 (19.08%) | 17827016 (64.84%) | 7142516 | 10684500 (38.86%) |
| 100222 | 6 | 1758(6) | Wean | 24913542 | 5853789 (23.50%) | 4319498 (17.34%) | 14740255 (59.17%) | 5434274 | 9305981 (37.35%) |
| 100222 | 7 | 1759(2) | Con | 22956789 | 5761556 (25.10%) | 4006293 (17.45%) | 13188940 (57.45%) | 6546383 | 6642557 (28.94%) |
| 100222 | 8 | 1759(3) | Con | 21469397 | 3958805 (18.44%) | 4070117 (18.96%) | 13440475 (62.60%) | 6732008 | 6708467 (31.25%) |
| 100225 | 1 | 1759(4) | Con | 8309401 | 2409626 (29.00%) | 1396116 (16.80%) | 4503659 (54.20%) | 1874430 | 2629229 (31.64%) |
| 100225 | 2 | 1759(6) | Con | 10953380 | 3589105 (32.77%) | 1645410 (15.02%) | 5718865 (52.21%) | 2485393 | 3233472 (29.52%) |
| 100225 | 3 | 1762(2) | Wean | 17219415 | 3569406 (20.73%) | 3263838 (18.95%) | 10386171 (60.32%) | 4666192 | 5719979 (33.22%) |
| 100225 | 4 | 1762(3) | Wean | 22511541 | 3744742 (16.63%) | 4362419 (19.38%) | 14404380 (63.99%) | 7611763 | 6792617 (30.17%) |
| 100225 | 6 | 1762(4) | Wean | 21814888 | 3756135 (17.22%) | 4117354 (18.87%) | 13941399 (63.91%) | 9381558 | 4559841 (20.90%) |
| 100225 | 7 | 1762(6) | Wean | 21543158 | 3703314 (17.19%) | 4283210 (19.88%) | 13556634 (62.93%) | 7248825 | 6307809 (29.28%) |
| 100225 | 8 | 1763(2) | Con | 21734946 | 3699559 (17.02%) | 4463100 (20.53%) | 13572287 (62.44%) | 7822737 | 5749550 (26.45%) |
| 100309 | 1 | 1763(3) | Con | 25761039 | 5210570 (20.23%) | 5015198 (19.47%) | 15535271 (60.31%) | 10601554 | 4933717 (19.15%) |
| 100309 | 2 | 1763(4) | Con | 28973074 | 5462311 (18.85%) | 6067176 (20.94%) | 17443587 (60.21%) | 10284606 | 7158981 (24.71%) |
| 100309 | 3 | 1763(6) | Con | 29314377 | 5567705 (18.99%) | 6088391 (20.77%) | 17658281 (60.24%) | 10681849 | 6976432 (23.79%) |
| 100309 | 4 | 1769(2) | Wean | 26630192 | 4520797 (16.98%) | 5119511 (19.22%) | 16989884 (63.80%) | 8763279 | 8226605 (30.89%) |
| 100309 | 6 | 1769(3) | Wean | 24587605 | 5183204 (21.08%) | 4772581 (19.41%) | 14631820 (59.51%) | 7857133 | 6774687 (27.55%) |
| 100309 | 7 | 1769(4) | Wean | 24104306 | 5103971 (21.17%) | 4597262 (19.07%) | 14403073 (59.75%) | 7550714 | 6852359 (28.43%) |
| 100309 | 8 | 1769(6) | Wean | 26321704 | 4798460 (18.23%) | 5206822 (19.78%) | 16316422 (61.99%) | 8581174 | 7735248 (29.39%) |
| 100316 | 1 | 1770(2) | Wean | 24813826 | 4710674 (18.98%) | 5224214 (21.05%) | 14878938 (59.96%) | 8872678 | 6006260 (24.21%) |
| 100316 | 2 | 1770(3) | Wean | 27345930 | 4670117 (17.08%) | 5807108 (21.24%) | 16868705 (61.69%) | 9103730 | 7764975 (28.39%) |
| 100316 | 3 | 1770(4) | Wean | 27371688 | 5102727 (18.64%) | 5806024 (21.21%) | 16462937 (60.15%) | 9508313 | 6954624 (25.41%) |
| 100316 | 4 | 1770(6) | Wean | 21700024 | 5126967 (23.63%) | 4031807 (18.58%) | 12541250 (57.79%) | 6012884 | 6528366 (30.10%) |
| 100316 | 6 | 1789(2) | Con | 21848794 | 5425338 (24.83%) | 4010789 (18.36%) | 12412667 (56.81%) | 6728162 | 5684505 (26.02%) |
| 100316 | 7 | 1789(3) | Con | 25587368 | 4816759 (18.82%) | 5651481 (22.09%) | 15119128 (59.09%) | 8729209 | 6389919 (24.98%) |
| 100316 | 8 | 1789(4) | Con | 24325238 | 4560273 (18.75%) | 4763625 (19.58%) | 15001340 (61.67%) | 8783726 | 6217614 (25.56%) |
| 100409 | 1 | 1789(6) | Con | 18569808 | 3851904 (20.74%) | 3815774 (20.55%) | 10902130 (58.71%) | 6054730 | 4847400 (26.10%) |
| 100409 | 2 | 1802(2) | Con | 19768291 | 3889379 (19.67%) | 3658744 (18.51%) | 12220168 (61.82%) | 6527900 | 5692268 (28.79%) |
| 100409 | 3 | 1802(3) | Con | 18668849 | 3996147 (21.41%) | 3357507 (17.98%) | 11315195 (60.61%) | 5736113 | 5579082 (29.88%) |
| 100409 | 4 | 1802(4) | Con | 20172805 | 3780879 (18.74%) | 3622253 (17.96%) | 12769673 (63.30%) | 6602469 | 6167204 (30.57%) |
| 100409 | 6 | 1802(6) | Con | 20364927 | 3743401 (18.38%) | 3607275 (17.71%) | 13014251 (63.91%) | 7815146 | 5199105 (25.53%) |
| 100409 | 7 | 1773(2) | Wean | 22077884 | 4213821 (19.09%) | 4308400 (19.51%) | 13555663 (61.40%) | 7126812 | 6428851 (29.12%) |
| 100409 | 8 | 1773(3) | Wean | 22878153 | 3747957 (16.38%) | 4457866 (19.49%) | 14672330 (64.13%) | 5792572 | 8879758 (38.81%) |
| 100421 | 1 | 1773(4) | Wean | 16256030 | 3560915 (21.91%) | 3011442 (18.53%) | 9683673 (59.57%) | 4454140 | 5229533 (32.17%) |
| 100421 | 2 | 1773(6) | Wean | 21827470 | 4003868 (18.34%) | 4336738 (19.87%) | 13486864 (61.79%) | 6479381 | 7007483 (32.10%) |
| 100421 | 3 | 1803(2) | Con | 23087721 | 4549405 (19.70%) | 4278948 (18.53%) | 14259368 (61.76%) | 7838335 | 6421033 (27.81%) |
| 100421 | 4 | 1803(3) | Con | 20985054 | 4247202 (20.24%) | 3946304 (18.81%) | 12791548 (60.96%) | 6643163 | 6148385 (29.29%) |
| 100421 | 6 | 1803(4) | Con | 21954247 | 4514804 (20.56%) | 4053299 (18.46%) | 13386144 (60.97%) | 7203517 | 6182627 (28.16%) |
| 100421 | 7 | 1803(6) | Con | 22488443 | 4575594 (20.35%) | 4270961 (18.99%) | 13641888 (60.66%) | 7176634 | 6465254 (28.75%) |
| 100421 | 8 | 1809(2) | Wean | 21152860 | 4480619 (21.18%) | 4099933 (19.38%) | 12572308 (59.44%) | 6797783 | 5774525 (27.29%) |
| 100728 | 1 | 1809(3) | Wean | 23718729 | 5774172 (24.34%) | 3918368 (16.52%) | 14026189 (59.14%) | 7575608 | 6450581 (27.19%) |
| 100728 | 3 | 1809(4) | Wean | 25733471 | 5750247 (22.35%) | 4444580 (17.27%) | 15538644 (60.38%) | 8011020 | 7527624 (29.25%) |
| 100728 | 8 | 1809(6) | Wean | 21896308 | 5250611 (23.98%) | 3496640 (15.97%) | 13149057 (60.05%) | 7659087 | 5489970 (25.10%) |
| **Average** | | | | 22692842 | 4479044 | 4325899 | 13887900 | 7402570 | 6485330 |
