# Supplementary material for: Transcriptomic analysis of the stress response to weaning at housing in bovine leukocytes using RNA-seq technology
Source: BMC Genomics. 2012 Jun 18;13:250. doi: 10.1186/1471-2164-13-250 (PMC3583219; doi:10.1186/1471-2164-13-250)
Supplement: Additional file 4 — Table S4.Significantly differentially expressed pathways between weaned and control calves. [file 1471-2164-13-250-S4.doc]

| **Table S4. Significantly differentially expressed pathways between weaned and control calves.** | | | |
| --- | --- | --- | --- |
|  | **Day post weaning** | | |
| **Pathway** | **Day 1** | **Day 2** | **Day 7** |
| **Cytokine signalling** | CCL24, CXCL5, FLT3, IFNK, CXCL8, KDR, CXCL7, TNFRSF11A, XCL2 |  |  |
| **Transmembrane transport** | **AQP1**, SLC10A6, SLC12A3, SLC14A1, SLC26A3, SLC4A5, SLC5A11, **SLC6A5** | **SLC12A3**, SLC24A5, SLC26A3, SLC29A4, SLC29A10, SLC4A1, SLC5A7, SLC6A15, SLC6A2 |  |
| **Haemostasis** | MRVI1, PDE5A | **COL1A1**, **COL1A2** | **COL1A1**, **COL1A2**, α2β1 |
| **GPRC signalling** |  | **ADRB1**, **ADRB3**, HTR1B, **GLP1R**, **PDE4C** | GNAI1, RGS1, RGS13, RGS17, **RGS20** |
| RED indicates genes up-regulated in weaned calves versus control calves; **GREEN** indicates genes down-regulated in weaned calves versus control calves.  Genes are listed if two criteria are met: 1) they are significantly differentially expressed (fold change ≥ 2 and false discovery rate (FDR) < 0.05); 2) the pathway is significantly differentially expressed as identified by GOseq and InnateDB (FDR < 0.1). | | | |
