# Supplementary material for: Transcriptomic analysis of the stress response to weaning at housing in bovine leukocytes using RNA-seq technology
Source: BMC Genomics. 2012 Jun 18;13:250. doi: 10.1186/1471-2164-13-250 (PMC3583219; doi:10.1186/1471-2164-13-250)
Supplement: Additional file 6 — Table S6.Significantly differentially expressed pathways in weaned calves. [file 1471-2164-13-250-S6.doc]

| **Table S6. Significantly differentially expressed pathways in weaned calves** | | | |
| --- | --- | --- | --- |
|  | **Day post weaning** | | |
| **Pathway** | **Day 1** | **Day 2** | **Day 7** |
| **Cytokine signalling** | **BMP6**, CCL2, CCL24, CXCL5, IL23R, IL5RA, CXCL8, LIFR, CXCL7, XCL2, AICDA, **COL1A1**, **COL1A2**, **IRS1** | **CCL19**, CXCL10, IL15RA, IL1A, IL23R, IL5RA, CXCL8, **MPL**, SKP1, PLCB4, TIAM2, AICDA, **COL1A1**, **COL1A2**, CCRL1 | IFNG, JAK2, LRPPRC, LIF |
| **Transmembrane transport** | SLC24A1, SLC24A5, **SLC22A1**, SLC22A7, SLC5A11, SLC5A7, SLC6A15, NUP54 | **CFTR**, **RHCG**, SLC1A1, SLC24A1, SLCA5, SLC2A13, SLC4A1, SLC5A7, SLC6A15, SLC6A2, SLC7A11, ABCA1, **ABCC11** |  |
| **Haemostasis** | **COL1A1**, **COL1A2**, α2β1, CPB2, PLAT, SERPINB2, ANGPT2, PDE3A, PLA2G4A, TRPC3 | **COL1A1**, **COL1A2**, α2β1, **COL3A1**, EDN1, PLA2G4A, TRPC6, **MPL**, P2RY12, TRPC3, IL1A, IL5RA, MME | **COL1A1**, **COL1A2**, **COL3A1**, α2β1, **ADCY8**, **EDNRB**, JAK2 |
| **GPRC signalling** | **ADM**, CALCRL | **ADM**, **ADRB3**, **CCL19**, CCRL1, CXCL10, EDN1, **FZD4**, **FZD7**, OXTR, P2RY12, P2RY14, TSHB, **VIPR1**, ADCY10, **EREG**, FGF2, FZD3, IL1A, CXCL8, NRG2, RGS1, RGS11, TSHB, PLCB4, TRPC3, TRPC6, CAMP, PLCB4, RYR3, **CFTR**, CHRNA2, GHSR, **NMUR2** | **ADCY8**, CD80, FZD4, FZD7, IFNG, **LIF**, **RAPGEF4**, RGS1, RGS2, **TAC3**, TSHB, **ADM**, ADRB2, CCL4, **EDNRB**, HTR2B, **UTS2** |
| RED indicates genes up-regulated versus Day 0; **GREEN** indicates genes down-regulated versus Day 0.  Genes are listed if two criteria are met: 1) they are significantly differentially expressed (fold change ≥ 2 and false discovery rate (FDR) < 0.05); 2) the pathway is significantly differentially expressed as identified by GOseq and InnateDB (FDR < 0.1). | | | |
