# Supplementary material for: Transcriptomic analysis of the stress response to weaning at housing in bovine leukocytes using RNA-seq technology
Source: BMC Genomics. 2012 Jun 18;13:250. doi: 10.1186/1471-2164-13-250 (PMC3583219; doi:10.1186/1471-2164-13-250)
Supplement: Additional file 7 — Table S7.Significantly differentially expressed pathways in control calves. [file 1471-2164-13-250-S7.doc]

| **Table S7. Significantly differentially expressed pathways in control calves.** | | | |
| --- | --- | --- | --- |
|  | **Day following weaning** | | |
| **Pathway** | **Day 1** | **Day 2** | **Day 7** |
| **Cytokine signalling** | IL13, **IL1A**, PDGFRB, **TNFRSF11A** | CCBP2, CCL22, **CCRL1** |  |
| **Transmembrane transport** |  | ABCA13, **ABCC2**, **ABCC8** |  |
| **Haemostasis** | GP1BA, **IL1A**, **MRVI1**, PLAT, PROC, **GNG12**, PDGFRB |  |  |
| **GPRC signalling** | PLAT, PROC, **SERPINA1**, IL13, **IL1A**, **WNT5B**, WNT8B | **C3AR1**, CCBP3, CCL22, **CCRL1**, GLP1R, **GNRH1** |  |
| RED indicates genes up-regulated in weaned calves versus control calves; **GREEN** indicates genes down-regulated in weaned calves versus control calves.  Genes are only listed if two criteria are met: 1) they are significantly differentially expressed (fold change ≥ 2 and false discovery rate (FDR) < 0.05); 2) the pathway is significantly differentially expressed as identified by GOseq and InnateDB (FDR < 0.1). | | | |
