# Supplementary material for: Transcriptomic analysis of the stress response to weaning at housing in bovine leukocytes using RNA-seq technology
Source: BMC Genomics. 2012 Jun 18;13:250. doi: 10.1186/1471-2164-13-250 (PMC3583219; doi:10.1186/1471-2164-13-250)
Supplement: Additional file 10 — Table S10.Significantly over-represented transcription factors based on transcription factor binding sites of up- and down-regulated genes between weaned and control calves. This file contains a table of transcription factors identified as having a role in the regulation of genes differentially expressed between weaned and control animals using oPOSSUM. [file 1471-2164-13-250-S10.doc]

| **Supplementary Table 10. Significantly over-represented transcription factors based on transcription factor binding sites of up- and down-regulated genes between weaned and control calves** | | | | | | | | |
| --- | --- | --- | --- | --- | --- | --- | --- | --- |
| **Treatment** | **Transcription factor** | **TF Class** | **No. submitted genes** | **No. included genes** | **Target gene hits** | **Target TFBS hits** | **Z-score** | **Fisher score** |
| **Weaned vs Control** |  |  |  |  |  |  |  |  |
| Day 1 | **NFKB1** | REL | 75 | 71 | 16 | 34 | 12.27 | 0.02442 |
| Day 2 | **SP1** | ZN-Finger, C2H2 | 94 | 87 | 64 | 286 | 10.27 | 0.008341 |
| Day 7 | Lhx3 | Homeo | 944 | 793 | 513 | 1868 | 36.1 | 0.00000000000906 |
|  | Foxd3 | Homeo | 944 | 793 | 536 | 2036 | 44.5 | 0.000000005882 |
|  | Prrx2 | Homeo | 944 | 793 | 673 | 6463 | 49.79 | 0.00000003235 |
|  | NKX3-1 | Homeo | 944 | 793 | 493 | 1607 | 31.43 | 0.00000009752 |
|  | **SP1** | ZN-Finger, C2H2 | 158 | 141 | 110 | 541 | 23.65 | 0.00001001 |
|  | SRY | HMG | 944 | 793 | 624 | 3916 | 37.47 | 0.00005745 |
|  | **MZF1 5-13** | ZN-Finger, C2H2 | 158 | 141 | 113 | 572 | 15.92 | 0.0001504 |
|  | Pdx1 | Homeo | 944 | 793 | 670 | 6552 | 47.06 | 0.0002094 |
|  | Nkx2-5 | Homeo | 944 | 793 | 681 | 7005 | 47.62 | 0.0002818 |
|  | Foxa2 | Forkhead | 944 | 793 | 494 | 1733 | 35.57 | 0.0004601 |
|  | Sox5 | HMG | 944 | 793 | 629 | 4126 | 32.0 | 0.0007285 |
|  | **REST** | ZN-Finger, C2H2 | 158 | 141 | 6 | 6 | 11.4 | 0.009661 |
| RED indicates analysis performed using up-regulated genes; **GREEN** indicates analysis performed using down-regulated genes | | | | | | | | |
