# Supplementary material for: Transcriptomic analysis of the stress response to weaning at housing in bovine leukocytes using RNA-seq technology
Source: BMC Genomics. 2012 Jun 18;13:250. doi: 10.1186/1471-2164-13-250 (PMC3583219; doi:10.1186/1471-2164-13-250)
Supplement: Additional file 11 — Table S11.Significantly over-represented transcription factors based on transcription factor binding sites of up- and down-regulated genes following weaning. This file contains a table of transcription factors identified as having a role in the regulation of genes differentially expressed following weaning using oPOSSUM. [file 1471-2164-13-250-S11.doc]

| **Supplementary Table 11. Significantly over-represented transcription factors based on transcription factor binding sites of up- and down-regulated genes following weaning** | | | | | | | | |
| --- | --- | --- | --- | --- | --- | --- | --- | --- |
| **Treatment** | **Transcription factor** | **TF Class** | **No. submitted genes** | **No. included genes** | **Target gene hits** | **Target TFBS hits** | **Z-score** | **Fisher score** |
| **Weaned** |  |  |  |  |  |  |  |  |
| Day 0 vs 1 | SP1 | ZN-Finger, C2H2 | 58 | 53 | 43 | 230 | 25.33 | 0.001226 |
|  | Sox5 | HMG | 215 | 170 | 141 | 1057 | 15.69 | 0.005115 |
|  | SRY | HMG | 215 | 170 | 142 | 971 | 15.01 | 0.0005537 |
|  | Pdx1 | Homeo | 215 | 170 | 150 | 1556 | 14.9 | 0.001884 |
|  | Prrx2 | Homeo | 215 | 170 | 147 | 1500 | 14.77 | 0.001319 |
|  | NHLH1 | bHLH | 58 | 53 | 29 | 50 | 13.96 | 0.0001506 |
|  | Foxd3 | Forkhead | 215 | 170 | 117 | 474 | 13.7 | 0.001539 |
|  | Foxa2 | Forkhead | 215 | 170 | 118 | 415 | 12.17 | 0.0003255 |
|  | FOXI1 | Forkhead | 215 | 170 | 117 | 413 | 10.97 | 0.000663 |
|  | Lhx3 | Homeo | 215 | 170 | 118 | 429 | 10.06 | 0.000006085 |
| Day 0 vs 2 | Pdx1 | Homeo | 192 | 162 | 146 | 1511 | 14.61 | 0.0002197 |
|  | Prrx2 | Homeo | 192 | 162 | 143 | 1442 | 13.55 | 0.0001892 |
|  | Foxa2 | Forkhead | 192 | 162 | 115 | 403 | 11.93 | 0.00008997 |
|  | TEAD1 | TEA | 82 | 73 | 29 | 49 | 11.89 | 0.003366 |
|  | Lhx3 | Homeo | 192 | 162 | 117 | 427 | 11.53 | 0.0000002648 |
|  | Nkx2-5 | Homeo | 192 | 162 | 148 | 1576 | 11.09 | 0.0002961 |
|  | MEF2A | MADS | 192 | 162 | 80 | 166 | 10.4 | 0.0002961 |
| Day 0 vs 7 | FOXI1 | Forkhead | 237 | 200 | 137 | 467 | 13.09 | 0.0003402 |
|  | Lhx3 | Homeo | 237 | 200 | 88 | 158 | 11.18 | 0.00001208 |
| RED indicates analysis performed using up-regulated genes; **GREEN** indicates analysis performed using down-regulated genes | | | | | | | | |
