# Supplementary material for: Transcriptomic analysis of the stress response to weaning at housing in bovine leukocytes using RNA-seq technology
Source: BMC Genomics. 2012 Jun 18;13:250. doi: 10.1186/1471-2164-13-250 (PMC3583219; doi:10.1186/1471-2164-13-250)
Supplement: Additional file 12 — Table S12.Significantly over-represented transcription factors based on transcription factor binding sites of up- and down-regulated genes following housing. This file contains a table of transcription factors identified as having a role in the regulation of genes differentially expressed following housing stress using oPOSSUM. [file 1471-2164-13-250-S12.doc]

| **Supplementary Table 12. Significantly over-represented transcription factors based on transcription factor binding sites of up- and down-regulated genes following housing** | | | | | | | | |
| --- | --- | --- | --- | --- | --- | --- | --- | --- |
| **Treatment** | **Transcription factor** | **TF Class** | **No. submitted genes** | **No. included genes** | **Target gene hits** | **Target TFBS hits** | **Z-score** | **Fisher score** |
| **Weaned** |  |  |  |  |  |  |  |  |
| Day 0 vs 2 | SP1 | ZN-Finger, C2H2 | 73 | 67 | 51 | 214 | 10.01 | 0.005796 |
| Day 0 vs 7 | **NKX3-1** | Homeo | 912 | 774 | 508 | 1688 | 33.86 | 0.0000000000007823 |
|  | **Lhx3** | Homeo | 912 | 774 | 506 | 1962 | 39.0 | 0.00000000000108 |
|  | **Foxd3** | Forkhead | 912 | 774 | 535 | 2055 | 40.72 | 0.00000000003793 |
|  | **Prrx2** | Homeo | 912 | 774 | 659 | 6765 | 53.08 | 0.00000001522 |
|  | **FOXI1** | Forkhead | 912 | 774 | 513 | 1723 | 27.93 | 0.00000002982 |
|  | **Foxg1** | Forkhead | 912 | 774 | 360 | 735 | 17.0 | 0.0000003186 |
|  | **Foxa2** | Forkhead | 912 | 774 | 504 | 1811 | 37.82 | 0.0000006219 |
|  | SP1 | ZN-finger, C2H2 | 160 | 145 | 115 | 606 | 38.41 | 0.000001446 |
|  | **HLF** | bZip | 912 | 774 | 329 | 543 | 14.13 | 0.000001766 |
|  | **Pdx1** | Homeo | 912 | 774 | 553 | 6837 | 49.8 | 0.000007135 |
|  | **MEF2A** | MADS | 912 | 774 | 337 | 635 | 14.14 | 0.0000095 |
|  | **Sox5** | HMG | 912 | 774 | 626 | 4326 | 35.07 | 0.00001499 |
|  | **SRY** | HMG | 912 | 774 | 611 | 4074 | 39.25 | 0.00003538 |
|  | USF1 | bHLH-ZIP | 160 | 145 | 103 | 313 | 14.65 | 0.00006722 |
|  | Mycn | bHLH-ZIP | 160 | 145 | 105 | 336 | 13.43 | 0.00008339 |
|  | **Nkz2-5** | Homeo | 912 | 774 | 688 | 7432 | 54.87 | 0.00009584 |
|  | Arnt | bHLH | 160 | 145 | 105 | 349 | 13.42 | 0.0001749 |
|  | Myf | bHLH | 160 | 145 | 93 | 232 | 10.76 | 0.0002006 |
|  | HNF4A | Nuclear Receptor | 160 | 145 | 78 | 131 | 11.96 | 0.000236 |
|  | MAX | bHLH-ZIP | 160 | 145 | 81 | 162 | 11.55 | 0.000236 |
|  | MZF1 5-13 | ZN-finger, C2H2 | 160 | 145 | 115 | 641 | 30.55 | 0.0002943 |
|  | NHLH1 | bHLH | 160 | 145 | 62 | 110 | 12.74 | 0.0007532 |
|  | **IRF1** | TRP-Cluster | 912 | 774 | 279 | 455 | 13.49 | 0.0008395 |
|  | SRF | MADS | 160 | 145 | 16 | 19 | 13.14 | 0.001503 |
| RED indicates analysis performed using up-regulated genes; **GREEN** indicates analysis performed using down-regulated genes | | | | | | | | |
